# Impairment of Vocal Expression of Negative Emotions in Patients with Alzheimer’s Disease

**DOI:** 10.3389/fnagi.2014.00101

**Published:** 2014-05-26

**Authors:** Kyung-Hun Han, Yuliya Zaytseva, Yan Bao, Ernst Pöppel, Sun Yong Chung, Jong Woo Kim, Hyun Taek Kim

**Affiliations:** ^1^Behavioral Science Research Center, Korea University, Seoul, South Korea; ^2^Human Science Center, Institute of Medical Psychology, Ludwig-Maximilians-Universität, Munich, Germany; ^3^Parmenides Center for Art and Science, Pullach im Isartal, Germany; ^4^Moscow Research Institute of Psychiatry, Moscow, Russia; ^5^Prague Psychiatric Center, 3rd Faculty of Medicine, Charles University in Prague, Prague, Czech Republic; ^6^Key Laboratory of Machine Perception, Department of Psychology, Peking University, Peking, China; ^7^Department of Oriental Neuropsychiatry, Medical College, KyungHee University, Seoul, South Korea; ^8^Department of Psychology, Korea University, Seoul, South Korea

**Keywords:** Alzheimer’s disease, emotional expression, vocal expression, autobiographical memory, episodic memory

## Abstract

Vocal expression of emotions (EE) in retrieval of events from autobiographical memory was investigated in patients in early stages of Alzheimer’s disease (AD). Twenty-one AD patients and 19 controls were interviewed, and EE of the reported memories was rated by 8 independent evaluators. The AD group had lower EE of both recent and remote memory than controls, although EE in remote memories was better preserved in both groups. We observed positive correlations between EE and indicators of cognitive competence in AD patients. AD Patients are impaired in the ability to express emotions already at early stages of the disease, and EE seems to deteriorate along with the progression of cognitive impairment.

## Introduction

Expression and recognition of emotions are essential for social communication. It is well established that emotions enhance episodic and autobiographical memory (Tulving, 1987); this functional relationship between memory and emotions is supported by observations indicating that memories for events can be intensified by strong emotional valence (Bower, 1992), and the recall is commonly better for emotionally colored autobiographical events (Holmes, 1970; Brewer, 1988; Wang, 2008; Rasmussen and Berntsen, 2009). Autobiographical memory is not only the encoding and retrieval of sets of information, but also remembering one’s own past event in its integrative detail grasping particular objects, emotions or feelings, thoughts, and senses related to that event.

Memory impairment is a hallmark of Alzheimer’s disease (AD). The difficulty in encoding and/or retrieval of new information is claimed to be one of the earliest symptoms in patients with AD (Murphy et al., 2008; Leyhe et al., 2009; Irish et al., 2011a; Bastin et al., 2012). It is known that remote memories in AD are better preserved than the recent ones (Sartori et al., 2004; Leyhe et al., 2009). At the same time, emotion processing in AD patients appears to be altered which to a certain degree influence negatively the quality of patient’s interaction with others (Bucks and Radford, 2004). The recent study of Irish et al. (2011a) reported the disruption of emotional re-experiencing in retrieval of recent autobiographical memories in AD patients.

Emotions are recognized and expressed in several ways: non-verbally using facial expression and gestures and verbally or vocally; in the latter case the prosody of language, its intonation pattern, its rhythmic structure, and speech velocity are commonly used to index emotional processes (Berry, 1975; Bachorowski and Owren, 1995; Mozziconacci, 2002; Owren et al., 2005; Pöppel, 2010). During communication in humans, information about speaker’s emotional state is conveyed by the modulation of tone of the voice, namely emotional prosody. Explicit judgments about emotional states using emotional prosody evaluation are commonly used as a method for psychological therapy effectiveness in a variety of neurological and psychiatric diseases (van den Broek, 2004; Quadfliega et al., 2007).

Previous research on autobiographical memory and emotions, including studies on AD has been mainly focused on the recognition of emotions but rarely on emotional expression. Specifically, Burton and Kaszniak (2006) observed the emotional expression in AD patients recording facial muscle activities. They found small changes in the zygomatic activity in AD patients. Henry et al. (2009) reported mixed results on the lack of amplification of emotions and intact suppression of emotions in response to the emotional stimuli (watching comedy film segments) in AD patients indicating disrupted regulation of expressed emotions. However, to date there are no studies on the vocal emotional expression of autobiographical memory retrieval in AD even though it could be a critical cue of diagnosis and therapy.

In the present study, we investigated vocal expression of emotions (EE) in the retrieval of autobiographical memory in the early stage of AD. We hypothesize that the emotional expression of AD patients is expected to be lower than in healthy individuals of the same age. Since remote memory is better preserved in Alzheimer’s patients we expect that the expression of that memories will be more intense due to close relatedness to salient life events. As a general assumption, one could also predict that alterations in emotional expression develop in parallel with cognitive deterioration as emotional processing and cognition are densely linked (Pessoa, 2008). For verifying these hypotheses, we used an explicit evaluation of vocal expression of emotion in the retrieval of subject’s own autobiographical memory. Such method proved to be ecologically valid (Scherer, 2003). It allows rather to measure quality of memory’s retrieval than its quantity. Indeed, the analysis of subjects’ own past memory is difficult to interpret in terms of accuracy of responses as compared to other validated quantitative methods, however, we attempted to improve the validity of the method controlling for homogeneity of memory recalls, concentrating only on the negative memories retrieval. We followed the argument of Berntsen (2002) claiming that the emotionally negative memories like traumatic events contain more important information to the survival than positive memories.

## Materials and Methods

### Subjects

Twenty-one patient with AD (10M/11F, age = 61.84 ± 5.2 years; years of education = 9.19 ± 4.92) and 19 matched healthy elderly controls (9M/10F, age = 61.84 ± 3.74 years; mean education = 11.26 ± 3.89) participated in the study. All patients were selected on the basis of neurological examination, neuropsychological testing from Korean-Dementia Rating Scale (K-DRS) (Choi, 1998), Mini Mental State Examination-Korean (MMSE-K) (Kwon and Park, 1989) and CT/MRI scan. Patients were diagnosed with early stage of dementia (K-DRS >106; MMSE-K range = 20–27) within 69 years at the most in accordance to the criteria of National Institute of Neurological and Communicative Diseases and Stroke/Alzheimer’s Disease and Related Disorders Association (McKhann et al., 1984). Healthy elderly controls were volunteers recruited from the local community, and were all in good mental and physical health. We applied the following exclusion criteria: a history of alcoholism, epilepsy, psychiatric illnesses including depression, significant head injury for both groups. The study was approved by the ethic committee of the KyungHee University Medical Centre (KHNMC-OH-IRB 2009-015), and all subjects provided informed consent for study participation.

### Procedure

The subjects underwent autobiographical interview, which was purposely designed for measuring four categories of retrieval of recent (yesterday, last week, last month) and remote memories. No cues were used, thus subjects recalled memories freely. In the remote memory retrieval, subjects were asked to report the most impressive event from their childhood. Most subjects recalled the evacuation during the Korean War. As all subjects experienced the war in their childhood, those who did not spontaneously recall the event of Korean War were asked for it. For the recent memories, at first subjects were asked to retrieve any impressive event of the last month, last week, and yesterday. An MP3 player recorded verbal responses during the test.

As a first step, four different episodes of a subject’s retrieved memories from each category were selected by experimenters (four persons including the interviewer with Ph.D. degree in human biology), who did not participate in the further experiment. The contextually negative emotional events were chosen as the majority of subjects did not recall any positive emotional events during free recall procedure. The most vocally emotionally expressed parts were selected for the next step of the experiment. Each selected track contained the record of voice (neutral passages reporting participant’s everyday life) for 10 s followed by the pause (5 s) and 30 s of retrieval of negative experiences.

As a second step, eight evaluators (age = 27.75 ± 4.09, 4M/4F, three Europeans familiar with Korean language and five Koreans) were recruited to perform emotional expression (EE) rating.

Before the procedure, all evaluators were given a detailed instruction and underwent a training of emotional expression evaluation. The training was conducted by two educated instructors (graduated school students: one Korean and one European). The recorded tracks were played randomly (recent and remote memories mixed). After listening to each track evaluators subsequently rated the emotional intensity of subject’s voice on 0–2 rating scale: 0 – unrelated to emotion, 1 – related, 2 – highly related. While rating, evaluators were asked to subjectively create a mental synthesis of the prosody and tone of the voice, its intonation, the rhythmic structure and velocity of speech including crying and laughing sounds which in all cases proved to be possible. For all conditions, the evaluators were blind with respect to group membership.

### Statistical analysis

The data were analyzed using Statistical Package for the Social Science (SPSS version 12.0). Results were expressed as means and SD for demographic data and cognitive functions, and Median for memories scores. We used χ^2^-tests complemented with the effect sizes in order to analyze differences in the proportion of patients and controls in categories of emotional expression (yesterday, last week, last month, remote) and Wilcoxon test for within-group comparison of recent (composite scores for yesterday, last week, and last month) versus remote memories. The sum scores were calculated for each memory category (rating scores of all eight evaluators were summed up) as well cognitive scores in order to indicate emotional expression and cognitive functioning competences. Correlative analysis between vocal expression (recent and remote) and cognition test scores, which were derived from K-DRS (i.e., attention, initiation–perseveration, construction, conceptualization, and memory) was done using Spearman correlation coefficient. Inter-rater reliability was calculated using Fleiss Kappa coefficient for multiple raters.

## Results

Demographical and clinical data on patients, controls as well as evaluators group are presented in Table 1.

**Table 1:** **Subjects’ and evaluators’ demographic data**.

	Age in years (mean and SD)	Sex	Education in years (mean and SD)	MMSE-K scores (mean and SD)	K-DRS scores (mean and SD)
Controls (*n* = 19)	61.84 ± 3.74	Male: 9	11.26 ± 3.89	27.89 ± 1.28*	135.52 + 4.09*
		Female: 10	
AD (*n* = 21)	61.84 ± 5.2	Male: 10	9.19 ± 4.92	23.19 ± 3.62	121.28 ± 9.74
		Female: 11	
Evaluators (*n* = 8)	27.75 ± 4.09	Male: 4	19.13 ± 1.89	–	–
		Female: 4	

*p < 0.01.

**Figure 1:**
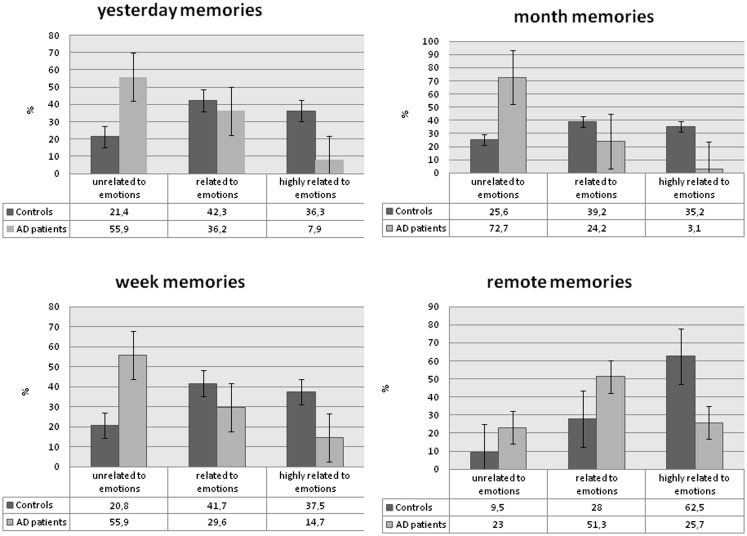
**Distribution of the percentages of the scores given in each memory category**. Comment: emotional expression of subject’s voice on 0–2 rating scale: 0 – unrelated to emotion, 1 – related, 2 – highly related.

A significantly higher proportion of AD patients’ memories was rated as 0–1 (not-related to emotions/related to emotions) whereas EE of controls were more likely to be rated as 1–2 (related to emotions/highly related to emotions) in all four categories of memories (χ^2^ = 34.09, *p* < 0.0001 with effect size *r* = 0.65 for yesterday memories; χ^2^ = 28.13, *p* < 0.0001, effect size *r* = 0.43 for week memories; χ^2^ = 53.06, *p* < 0.0001, *r* = 0.54 for month memories; χ^2^ = 27.82, *p* < 0.0001, *r* = 0.62 for remote memories) (Figure 1).

Taking into account the similar distribution of the scores in yesterday, last week, and last month memories, the scores of these categories were merged into recent memory score, which was used for further within-group analysis. In recent versus remote memories comparison, there was a higher emotional involvement in remote memory retrieval than in recent both in AD patients (*Z* = −3.36, *p* < 0.001) and in controls (*Z* = −2.48, *p* < 0.0039).

Explorative correlation analysis between the emotional vocal expression and five cognitive functions (attention, initiation-perseveration, construction, conceptualization, and memory) in overall participants sample revealed positive correlations between EE of all memory categories (Table 2).

**Table 2:** **A comparison between AD groups and healthy controls, and correlations between emotional expression and cognitive functions**.

Group	Values	Yesterday (controls = 19, AD = 21)	Week (controls = 19, AD = 17)	Month (controls = 18, AD = 8)	Remote (controls = 19, AD = 20)
Controls	Mean + SD	9.73 (±3.42)	10.05 (+3.53)	10.00 (+4.36)	13.15 (+2.65)
	Median	1	1	1	2
	25–75%	1–1.5	1–2	1–2	1.5–2
AD	Mean + SD	4.14 (±2.55)	5.52 (+4.75)	5.37 (+2.38)	8.15 (+3.54)
	Median	0	0	0.75	1
	25–75%	0–1	0–1	0.5–1	1–1

**K-DRS**	**Group**	**Mean (±SD)**	**Recent memory**		**Remote memory**	
			**Spearman *r***	***p***	**Spearman *r***	***p***

**CORRELATIONS BETWEEN EE AND COGNITIVE FUNCTIONS**						
Attention	Controls	35.68 (±0.88)*	0.491**	0.001	0.412**	0.009
	AD	33.95 (±2.63)	
Initiation/perseveration	Controls	33.73 (±3.39)	0.452**	0.003	0.295	0.068
	AD	31.42 (±5.08)	
Construction	Controls	5.89 (±0.315)*	0.330*	0.037	0.379*	0.017
	AD	5.05 (±1.49)	
Conceptualization	Controls	36.73 (±1.72)**	0.412**	0.008	0.437**	0.005
	AD	33.85 (±3.96)	
Memory	Controls	23.47 (±1.54)**	0.700**	0.000	0.635**	0.000
	AD	16.95 (±3.52)	
Total	Controls	135.52 (±4.91)**	0.672**	0.000	0.459**	0.003
	AD	121.28 (±9.74)	
MMSE-K	Controls	27.89 (±1.28)**				
	AD	23.19 (±3.62)	

Comment: correlative analysis was done in overall groups, *p < 0.05, **p < 0.01.

The reliability of agreement results are as follows: for yesterday memories (FK = 0.1922, SE = 0.02, 95% CI = 0.1501–0.2344), for week memories (FK = 0.5069 SE = 0.0225, 95% CI = 0.4628–0.5510), for month memories (FK = 0.2635 SE = 0.0224, 95% CI = 0.2196–0.3074), for remote memories (FK = 0.3571 SE = 0.0213, 95% CI = 0.3154–0.3988).

## Discussion

The present study yielded several new results. Firstly, we showed that patients with early stages of AD are impaired in the ability to express their emotions during autobiographical memory retrieval both of recent and remote memories. Previous research was focused primarily on non-verbal emotional expression in AD patients (Smith, 1995; Seidl et al., 2012), and it was performed in already severely cognitively impaired patients with AD. For the first time, we report that low vocal emotional expression is already present at an early stage of AD. In contrast to other studies, we explored emotional expressions referring to autobiographical memories rather than to semantic knowledge. Episodic experiences stored in autobiographical memory are essential to construct and to maintain a sense of personal identity and self-continuity (Piolino et al., 2009; Pöppel, 2010). Based on our findings one can conclude that changes in emotional expression might also affect self processing.

Even though the AD group showed a lower emotional expression for recent and remote events compared to the control group, the emotional expression when recalling remote memories were better preserved. These findings confirm previous results which indicated that at early stages AD patients exhibit difficulties in retrieving memories from the time after the AD’s onset, but are capable of retrieving memory from long time ago (Kaszniak et al., 1993; Clare et al., 2000). This might match other observations of an interaction between age and life span (Botwinick and Storandt, 1980; Perlmutter et al., 1980). Botwinick and Storandt (1980) suggested that the individuals recall those events better that happened with them when they were young adults (aged 15–25) compared to the events from the other life time period. In the current study, all subject recalled the events related to Korean War as remote memory, which they experienced as the teenagers.

Furthermore, we found that the level of emotional expression competence corresponds to the cognitive functioning. As cognitive deterioration determines dementia diagnosis, and the incremental decrease of cognitive functions is followed by the declining of the emotional expression, therefore EE might be considered as a substantial parameter in the diagnostic process.

Bucks and Radford (2004) reported that AD patients have deficit of emotional processing and cognitive functioning compared to healthy elderly adults. However, other studies (Koff et al., 1999; Lavenu et al., 1999; Han and Pöppel, 2009) have argued that a deficit in emotional processing is secondary to cognitive impairments associated with AD. For example, Koff et al. (1999) indicated that AD patients do not have a primary deficit in the processing of emotions. However, all the previous studies on emotional processing in AD used the emotional recognition tasks and identification of basic emotions. The facial expressions decrease gradually with the progression of dementia (Norberg et al., 1986) and inappropriate facial expression is present at the early stage of AD (Kunz et al., 2007). In our study, the vocal emotional expression was evaluated and appeared to be impaired in early stage of AD. It may suggest that the quality of the retrieval of autographical memory is generally declined in early stage of AD.

Furthermore, recently López-de-Ipiña et al. (2013) reported gradual changes in the spontaneous speech and emotional response in AD patients similar to the results of Kertesz and Clydesdale (1994) who observed the decline in expressive language skill in AD. As we have demonstrated, although the patients were able to recall emotional events from their own memory, they did not reproduce the events with a proper vocal expression. This finding go in line with the recent research of Irish et al. (2011b) suggesting that AD patients reproduce their own past events in fragmented and depersonalized way even though they recall evocative events.

As a limitation of the study, we report a small sample size and certain subjectivity of the external evaluation; one can predict the slight difference in scoring. However, the comparison did not reveal the substantial difference between the scores. Emotional expression in patients was more likely to be rated 0–1, while controls were mostly rated as 1–2.

In conclusion, frequent observations of caregivers suggest that AD patients in their early stages do not express sufficiently their emotions, demonstrating symptoms similar to depression and also altered communication or activity. Indeed, there are cases when such patients are misdiagnosed as being depressed. We showed here that emotional expression is affected in early stages of AD, and in patients who are not clinically diagnosed as depressed. Although, there has been an increasing interest in training of emotional expression, mainly non-verbal, our findings suggest that vocal emotional expression might be a target for therapeutic interventions.

## Conflict of Interest Statement

The authors declare that the research was conducted in the absence of any commercial or financial relationships that could be construed as a potential conflict of interest.

## References

[B1] Bachorowski J. A. Owren M. J. (1995). Vocal expression of emotion: acoustic properties of speech are associated with emotional intensity and context. Psychol. Sci. 6, 219–224 10.1111/j.1467-9280.1995.tb00596.x

[B2] Bastin C. Feyers D. Jedidi H. Bahri M. A. Degueldre C. Lemaire C. (2012). Episodic autobiographical memory in amnestic mild cognitive impairment: what are the neural correlates? Hum. Brain Mapp. 34, 1811–1825 10.1002/hbm.22032 22422512 PMC6869917

[B3] Berntsen D. (2002). Tunnel memories for autobiographical events: central details are remembered more frequently from shocking than from happy experiences. Mem. Cognit. 30, 1010–1020 10.3758/BF03194319 12507366

[B4] Berry K. K. (1975). Developmental study of recognition of antecedents of infant vocalizations. Percept. Mot. Skills 41, 400–402 10.2466/pms.1975.41.2.400

[B5] Botwinick J. Storandt M. (1980). Recall and recognition of old information in relation to age and sex. J. Gerontol. 35, 70–76 10.1093/geronj/35.1.70 7350223

[B6] Bower G. H. (1992). “How might emotions affect learning?,” in The Handbook of Emotion and Memory: Research and Theory, ed. Christianson S. A. (Hillsdale, NJ: Lawrence Erlbaum), 3–31

[B7] Brewer W. F. (1988). “Memory for randomly sampled autobiographical events,” in Remembering Reconsidered: Ecological and Traditional Approaches to the Study of Memory. Emory Symposia in Cognition, 2nd Edn, eds Neisser U. Winograd E. (New York, NY: Cambridge University Press), 21–90

[B8] Bucks R. S. Radford S. A. (2004). Emotion processing in Alzheimer’s disease. Aging Ment. Health 8, 222–232 10.1080/13607860410001669750 15203403

[B9] Burton K. W. Kaszniak A. W. (2006). Emotional experience and facial expression in Alzheimer’s disease. Neuropsychol. Dev. Cogn. B Aging Neuropsychol. Cogn. 13, 636–651 10.1080/13825580600735085 16887793

[B10] Choi J. Y. (1998). Professional Manuel of Korean Version – Dementia Rating Scale (K-DRS). Seoul: Hakjisa

[B11] Clare L. Wilson L. B. Carter G. Breen K. Gosses A. Hodges J. R. (2000). Intervening with everyday memory problems in dementia of Alzheimer type: an errorless learning approach. J. Clin. Exp. Neuropsychol. 22, 132–146 10.1076/1380-3395(200002)22:1;1-8;FT132 10649552

[B12] Han K. H. Pöppel E. (2009). Analysis of the mental images in episodic memory with comparison between the patients with dementia of Alzheimer’s type and healthy elderly people. Korean J. Cogn. Sci. 20, 79–107

[B13] Henry J. D. Rendell P. G. Scicluna A. Jackson M. Phillips L. H. (2009). Emotion experience, expression, and regulation in Alzheimer’s disease. Psychol. Aging 24, 252–257 10.1037/a0014001 19290761

[B14] Holmes D. S. (1970). Differential change in affective intensity and the forgetting of unpleasant personal experiences. J. Pers. Soc. Psychol. 15, 234–239 10.1037/h0029394 5485415

[B15] Irish M. Hornberger M. Lah S. Miller L. Pengas G. Nestor P. J. (2011a). Profiles of recent autobiographical memory retrieval in semantic dementia, behavioural-variant frontotemporal dementia, and Alzheimer’s disease. Neuropsychologia 49, 2694–2702 10.1016/j.neuropsychologia.2011.05.017 21658396

[B16] Irish M. Lawlor B. A. O’Mara S. M. Coen R. F. (2011b). Impaired capacity for autonoetic reliving during autobiographical event recall in mild Alzheimer’s disease. Cortex 47, 236–249 10.1016/j.cortex.2010.01.002 20153463

[B17] Kaszniak A. W. DiTraglia G. Trosset M. W. (1993). Self-awareness of cognitive deficit in patients with probable Alzheimer’s disease. J. Clin. Exp. Neuropsychol. 15, 30

[B18] Kertesz A. Clydesdale S. (1994). Neuropsychological deficits in vascular dementia vs Alzheimer’s disease: frontal lobe deficits prominent in vascular dementia. Arch. Neurol. 51, 1226–1231 10.1001/archneur.1994.00540240070018 7986178

[B19] Koff E. Zaitchik D. Montepare J. Albert M. S. (1999). Emotion processing in the visual and auditory domains by patients with Alzheimer’s disease. J. Int. Neuropsychol. Soc. 5, 32–40 9989022 10.1017/s1355617799511053

[B20] Kunz M. Scharmann S. Hemmeter U. Schepelmann K. Lautenbacher S. (2007). The facial expression of pain in patients with dementia. Pain 133, 221–228 10.1016/j.pain.2007.09.007 17949906

[B21] Kwon Y. C. Park J. H. (1989). Standardizing research of mini-mental state examination-Korean (MMSE-K). J. Korean Neuropsychiatric. Assoc. 28, 125–135

[B22] Lavenu I. Pasquier F. Lebert F. Petit H. Van der Linden M. (1999). Perception of emotion in frontotemporal dementia and Alzheimer disease. Alzheimer Dis. Assoc. Disord. 13, 96–101 10.1097/00002093-199904000-00007 10372953

[B23] Leyhe T. Müller S. Milian M. Eschweiler G. W. Saur R. (2009). Impairment of episodic and semantic autobiographical memory in patients with mind cognitive impairment and early Alzheimer’s disease. Neuropsychologia 47, 2464–2469 10.1016/j.neuropsychologia.2009.04.018 19409401

[B24] López-de-Ipiña K. Alonso J. B. Travieso C. M. Solé-Casals J. Egiraun H. Faundez-Zanuy M. (2013). On the selection of non-invasive methods based on speech analysis oriented to automatic Alzheimer disease diagnosis. Sensors (Basel) 13, 6730–6745 10.3390/s130506730 23698268 PMC3690078

[B25] McKhann G. Drachman D. Folstein M. Katzman R. Price D. Stadlan E. M. (1984). Clinical diagnosis of Alzheimer’s disease: report of the NINCDS-ADRDA Work Group under the auspices of department of health and human services task force on Alzheimer’s disease. Neurology 34, 939–944 10.1212/WNL.34.7.939 6610841

[B26] Mozziconacci S. J. L. (2002). “Prosody and emotions,” in Proceedings of the 1st International Conference on Speech Prosody (Aix-en-Provence), 1.9

[B27] Murphy K. J. Troyer A. K. Levine B. Moscovitch M. (2008). Episodic, but not semantic, autobiographical memory is reduced in amnestic mild cognitive impairment. Neuropsychologia 46, 3116–3123 10.1016/j.neuropsychologia.2008.07.004 18675285 PMC2629588

[B28] Norberg A. Melin E. Asplund K. (1986). Reactions to music touch and object presentation in the final stage of dementia. An exploratory study. Int. J. Nurs. Stud. 23, 315–323 10.1016/0020-7489(86)90054-4 3536774

[B29] Owren M. J. Rendall D. Bachorowski J. A. (2005). “Conscious and unconscious emotion in nonlinguistic vocal communication,” in Emotion and Consciousness, eds Barrett L. F. Niedenthal P. M. Winkielman P. (New York, NY: Guilford), 185–204

[B30] Perlmutter M. Metzger R. Miller K. Nezworski T. (1980). Memory of historical events. Exp. Aging Res. 6, 46–60 10.1080/03610738008258345 7379833

[B31] Pessoa L. (2008). On the relationship between emotion and cognition. Nat. Rev. Neurosci. 9, 148–158 10.1038/nrn2317 18209732

[B32] Piolino P. Desgranges B. Eustache F. (2009). Episodic autobiographical memories over the course of time: cognitive, neuropsychological and neuroimaging findings. Neuropsychologia 47, 2214–2229 10.1016/j.neuropsychologia.2009.01.020 19524095

[B33] Pöppel E. (2010). “Perceptual identity and personal self,” in Personality from Biological, Cognitive, and Social Perspectives, eds Maruszewski T. Fajkowska M. Eysenck M. M. (Clinton Corners, NY: Eliot Werner Publications), 75–82

[B34] Quadfliega S. Wendtb B. Mohra A. Miltnera W. H. Straubea T. (2007). Recognition and evaluation of emotional prosody in individuals with generalized social phobia: a pilot study. Behav. Res. Ther. 45, 3096–3103 10.1016/j.brat.2007.08.003 17880917

[B35] Rasmussen A. S. Berntsen D. (2009). Emotional valence and the functions of autobiographical memories: positive and negative memories serve different functions. Mem. Cognit. 37, 477–492 10.3758/MC.37.4.477 19460954

[B36] Sartori G. Snitz B. E. Sorcinelli L. Daum I. (2004). Remote memory in advanced Alzheimer’s disease. Arch. Clin. Neuropsychol. 19, 779–789 10.1016/j.acn.2003.09.007 15288331

[B37] Scherer K. R. (2003). Vocal communication of emotion: a review of research paradigms. Speech Commun. 40, 227–256 10.1016/S0167-6393(02)00084-5

[B38] Seidl U. Lueken U. Thomann P. A. Kruse A. Schröder J. (2012). Facial expression in Alzheimer’s disease: impact of cognitive deficits and neuropsychiatric symptoms. Am. J. Alzheimers Dis. Other Demen. 27, 100–106 10.1177/1533317512440495 22495337 PMC10697354

[B39] Smith M. C. (1995). Facial expression in mild dementia of the Alzheimer type. Behav. Neurol. 8, 149–156

[B40] Tulving E. (1987). Multiple memory systems and consciousness. Hum. Neurobiol. 6, 67–80 3305441

[B41] van den Broek E. L. (2004). Emotional prosody measurement (EPM): a voice-based evaluation method for psychological therapy effectiveness. Stud. Health Technol. Inform. 103, 118–125 10.3233/978-1-60750-946-2-118 15747913

[B42] Wang Q. (2008). Emotion knowledge and autobiographical memory across the preschool years: a cross-cultural longitudinal investigation. Cognition 108, 117–135 10.1016/j.cognition.2008.02.002 18353299

